# A novel miRNA negatively regulates resistance to Glomerella leaf spot by suppressing expression of an *NBS* gene in apple

**DOI:** 10.1038/s41438-019-0175-x

**Published:** 2019-08-01

**Authors:** Yi Zhang, Qiulei Zhang, Li Hao, Shengnan Wang, Shengyuan Wang, Wenna Zhang, Chaoran Xu, Yunfei Yu, Tianzhong Li

**Affiliations:** Laboratory of Fruit Cell and Molecular Breeding, China Agricultural University, Beijing, 100193 China

**Keywords:** Plant immunity, Plant molecular biology

## Abstract

Glomerella leaf spot (GLS) of apple (*Malus*×*domestica* Borkh.), caused by *Glomerella cingulata*, is an emerging fungal epidemic threatening the apple industry. Little is known about the molecular mechanism underlying resistance to this devastating fungus. In this study, high-throughput sequencing technology was used to identify microRNAs (miRNAs) involved in GLS resistance in apple. We focused on miRNAs that target genes related to disease and found that expression of a novel miRNA, *Md-miRln20*, was higher in susceptible apple varieties than in resistant ones. Furthermore, its target gene *Md-TN1-GLS* exhibited the opposite expression pattern, which suggested that the expression levels of *Md-miRln20* and its target gene are closely related to apple resistance to GLS. Furthermore, downregulation of *Md-miRln20* in susceptible apple leaves resulted in upregulation of *Md-TN1-GLS* and reduced the disease incidence. Conversely, overexpression of *Md-miRln20* in resistant apple leaves suppressed *Md-TN1-GLS* expression, with increased disease incidence. We demonstrated that *Md-miRln20* negatively regulates resistance to GLS by suppressing *Md-TN1-GLS* expression and showed, for the first time, a crucial role for miRNA in response to GLS in apple.

## Introduction

Glomerella leaf spot (GLS) of apple (*Malus*×*domestica* Borkh.) is an emerging fungal epidemic that has caused great damage in China recently^1^. GLS, which is caused by *Glomerella cingulata*, was first reported in the US in 1970^2^ but was not noticed in China until 2011^3^. Today, GLS is an economically important disease that causes early severe defoliation, weakens tree vigor, and reduces apple production. As soon as 2 days after infection with GLS, reddish-purple spots appear on the leaves of infected plants. The spots rapidly expand and merge into necrotic lesions in ensuing days, after which the leaves often turn yellow and drop^4^. Fruits can also be infected, with small light-brown sunken lesions (1–5 mm in diameter), which do not increase in size over time^5^. In China, the varieties ‘Golden Delicious’, ‘Gala’, and ‘Qinguan’ are highly susceptible to GLS, whereas ‘Fuji’ and ‘Red Star’ are resistant. In apple, resistance to GLS is controlled by a single recessive gene^6^, and the location of a GLS resistance gene locus (*R*_*gls*_) has been identified by bulked segregant analysis (BSA)^7,8^.

MicroRNAs (miRNAs) are small noncoding RNA molecules that exert regulatory functions by binding to complementary sequences in mRNAs to promote degradation or inhibit translation, resulting in silencing of the corresponding genes^9^. miRNAs play a crucial role in almost all biological processes in plants, including growth and development, hormone signaling, and stress responses^10,11^. Mounting evidence has revealed that miRNAs play a significant role in the response of plants to pathogens by directly and indirectly regulating expression of resistance (*R*) genes^12,13^. *R* genes encode a class of protein receptors, most of which contain a nucleotide-binding site (NBS) and one or more leucine-rich repeat (LRR) domains that recognize virulence effectors, leading to effector-triggered immunity (ETI)^14,15^. For example, members of the potato (*Solanum tuberosum*) miR482 superfamily target a class of disease resistance proteins with NBS and LRR motifs. *Verticillium dahliae* injection suppresses expression of miR482e and upregulates several NBS–LRR targets of miR482e, and transgenic potato plantlets overexpressing miR482e show hypersensitivity to *V. dahliae* infection^16^. Similarly, *Soybean mosaic virus* (SMV) counteracts soybean (*Glycine max*) defense responses through downregulation of several NBS–LRR family resistance genes by inducing accumulation of miR1507a and miR1507c^17^. In ‘Golden Delicious’ apple, suppression of five *R* genes, which are targeted by mdm-siR277-1 and mdm-siR277-2, results in susceptibility to *Alternaria* leaf spot fungus (ALT1)^13^. These findings highlight the fundamental role of miRNAs in plant immunity.

To date, research regarding GLS of apple has mainly focused on isolating and identifying the pathogen^18^, environmental conditions affecting infection and spread^1^, the disease mechanism^19^, control methods^3^, and genetic mapping of GLS resistance gene loci^6–8^. However, little is known about the molecular mechanism underlying the plant’s response to GLS infection.

In this study, we used high-throughput sequencing to identify miRNAs involved in apple resistance to GLS. We found that expression of a novel miRNA named *Md-miRln20* was higher in susceptible than in resistant apple varieties, whereas its target gene, named *Md-TN1-GLS*, showed the opposite trend. These findings indicate that expression of *Md-miRln20* and its target gene is closely related to GLS resistance in apple. Further analysis demonstrated that *Md-miRln20* is able to regulate resistance to GLS by suppressing *Md-TN1-GLS* expression. These results illustrate for the first time the crucial role of miRNA in the response of apple to GLS.

## Results

### Resistance evaluation of apple germplasms to GLS

**Fig. 1:**
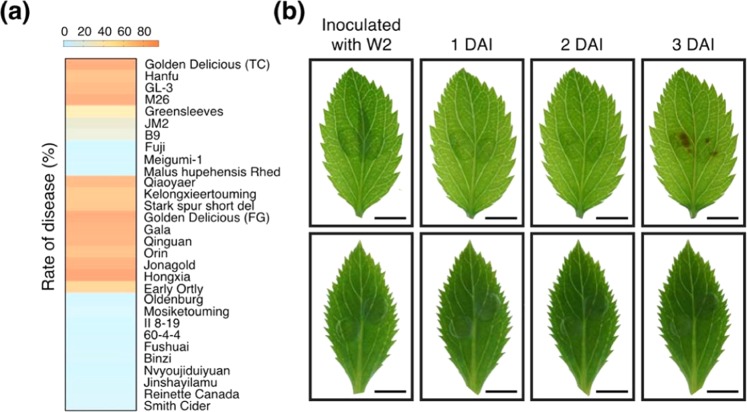
Resistance evaluation of apple germplasms to Glomerella leaf spot and small RNA sequencing of the leaves of susceptible ‘Golden Delicious’. **a** The incidence index of apple germplasms. The first 10 were tissue-cultured (TC), and the last 20 were field-grown (FG). **b** Symptoms of the leaves of susceptible (upper) and resistant (lower) apple germplasms after inoculation with the W2 strain of *G. cingulata*. Bar = 0.5 cm

A spore suspension of *G. cingulata* strain W2 (W2) was inoculated onto detached leaves of 30 different apple germplasms, of which 10 were tissue cultured and 20 grown in the field, along with three F_1_ hybrid groups (Table S1; Figs. S1 and S2). On the inoculated leaves of susceptible apple germplasms, necrotic lesions became visible and expanded rapidly from 2 DAI; no changes were observed on the leaves of resistant germplasms (Fig. 1b). The incidence of GLS was calculated at 3 DAI. Four of the 10 tissue-cultured apple germplasms showed high disease rates, including ‘Golden Delicious’, ‘Hanfu’, ‘GL-3’, and ‘M26’. ‘Fuji’, ‘Meigumi-1’ (a self-crossed progeny of ‘Meigumi’), and ‘*Malus hupehensis* Rhed’ were almost completely immune to GLS (Figs. 1a, S1a, and S1b). We also used an SSR marker (S0405127)^8^ to further evaluate resistance, and a smaller band, 330 bp in size, was found only for susceptible plants. The results of the SSR analysis were essentially in agreement with the incidence of GLS (Figs. 1a and S1). The genotype of the resistant plants was rr, and the genotypes of the susceptible plants were RR and Rr^6^. ‘Nagafu 2’ and ‘Ryoka no kisetsu’ were resistant, with a genotype of rr. ‘Golden Delicious’, ‘62-45’, ‘Hanfu’, and ‘Yueshuai’ were susceptible, and each carried at least one R allele (Fig. S2). The separation ratios of susceptible-to-resistant plants in the three F_1_ hybrid groups we analyzed (‘Golden Delicious’ × ‘Nagafu 2’, ‘Hanfu’ × ‘Yueshuai’, and ‘62-45’ × ‘Ryoka no kisetsu’) were statistically consistent with the theoretical ratios of 1:1, 3:1, and 1:0 (Figs. S2 and S3; Table S2). These data verified that resistance to GLS is controlled by a single recessive gene in apple and indicated the genotypes ‘Golden Delicious’, ‘Hanfu’, ‘Yueshuai’, and ‘62-45’ to be Rr, Rr, Rr, and RR, respectively^6–8^.

### High-throughput sequencing of small RNAs and prediction of their targets in GD leaves infected with *Glomerella cingulata*

To identify miRNAs that respond to GLS in apple, we constructed a small RNA library from W2-inoculated leaves of ‘Golden Delicious’ (GD) collected at 3 DAI (Fig. 1b). A total of 10,096,436 raw reads were generated by Illumina high-throughput sequencing. After removing low-quality reads, 3′ and 5′ adaptors, and reads shorter than 18 nt and also filtering out other contaminant reads, 9,752,888 reads remained (Table S3). Unique sequences of 18–25 nt were mapped to the mature and precursor sequences of miRNAs from other plant species available in miRBase 21 (http://www.mirbase.org/)^20^. Based on this analysis, we identified 50 known and 101 novel miRNAs (Tables S4 and S5). To better understand the biological functions of the identified novel miRNAs, psRobot software was employed to predict their target genes^21^. According to target gene annotations in NCBI (https://www.ncbi.nlm.nih.gov), we found eight miRNAs likely to be involved in defense-related processes (Table 1).

**Table 1:** Novel miRNAs and predicted targets chosen for expression analysis

miRNA	Gene ID	Target description
Md-miRln2	MDP0000277868	TMV resistance protein N-like
Md-miRln8	MDP0000935996 (MD11G1059400)	Probable WRKY transcription factor 26
Md-miRln14	MDP0000610304 (MD02G1052800)	Probable WRKY transcription factor 16
Md-miRln20	MDP0000234409 (MD02G1112700)	TMV resistance protein N-like
Md-miRln29	MDP0000310092 (MD07G1205300)	Putative disease resistance protein At1g52660
Md-miRln30	MDP0000184270 (MD05G1025200)	Disease resistance protein RPM1-like
Md-miRln33	MDP0000382588 (MD02G1165600)	Probable disease resistance protein At5g66910
Md-miRln34	MDP0000214891	TMV resistance protein N-like

### Expression analysis of potential GLS-responsive novel miRNA-target pairs

**Fig. 2:**
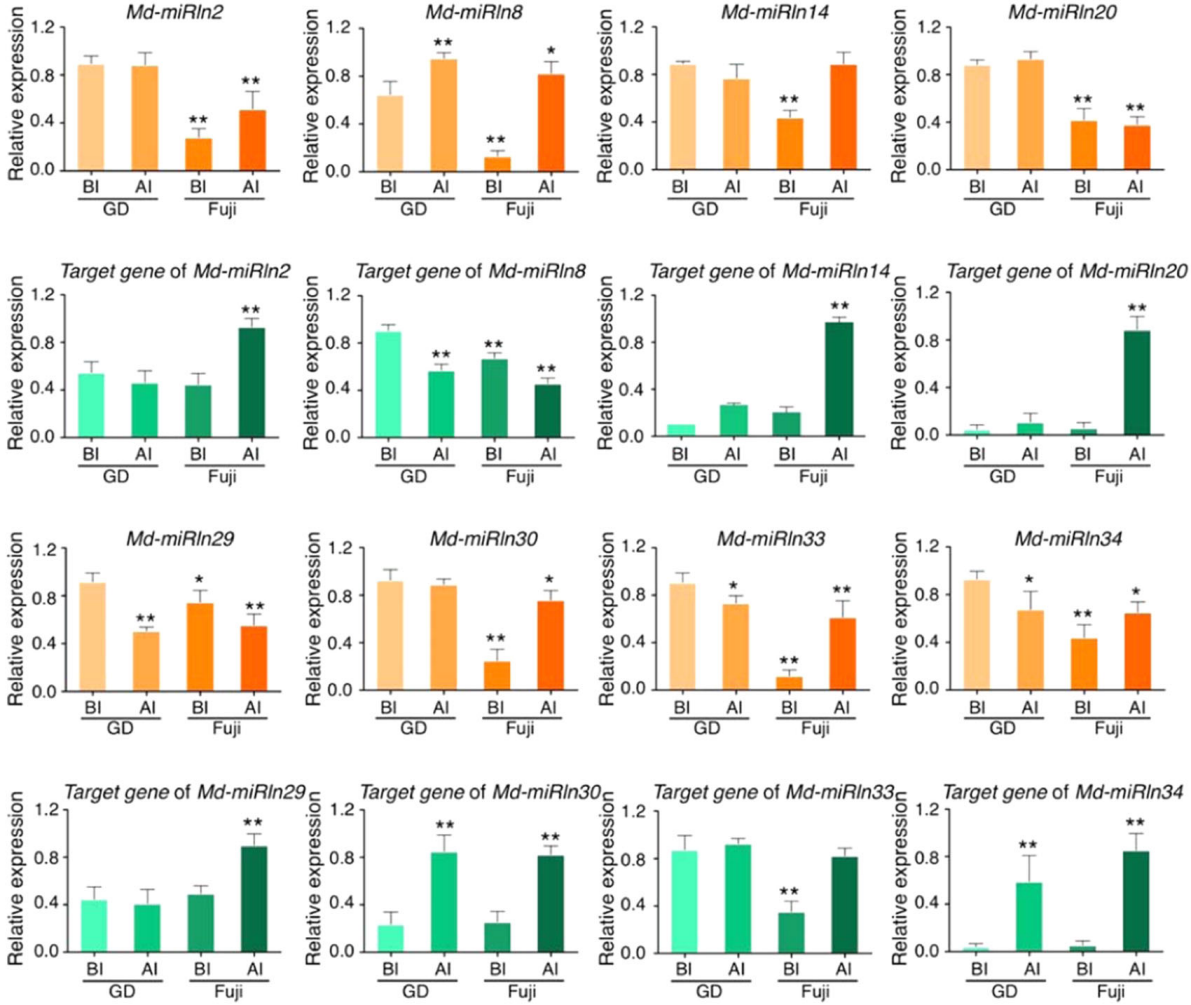
Expression of novel miRNAs and their predicted targets in leaves of GD and ‘Fuji’. RT-qPCR analysis of leaves before inoculation (BI) and 3 days after inoculation (AI) with W2. The orange bars indicate expression of miRNAs, and the green bars indicate that of their target genes. Error bars represent standard deviations calculated from three biological replicates. Student’s *t*-test: *P < 0.05 and **P < 0.01

To uncover novel miRNAs that respond to GLS, we analyzed in GD (susceptible variety) and ‘Fuji’ (resistant variety) expression of eight miRNAs and their target genes (Table 1) related to defense according to annotations in NCBI. Notably, *Md-miRln20* expression was approximately twice as high in GD as in ‘Fuji’, both before and after W2 inoculation. Moreover, expression of the corresponding target gene *MDP0000234409* was significantly upregulated after inoculation in ‘Fuji’ but remained low in GD (Fig. 2). These results show that *Md-miRln20* and its target gene likely play a role in the response of apple to GLS.

### Expression levels of *Md-miRln20* and its target gene after inoculation are associated with GLS resistance in apple

*Md-miRln20* was expressed at higher levels in the susceptible GD variety than in the resistant ‘Fuji’ variety, and its target gene was upregulated only in ‘Fuji’ after inoculation. Thus, we next examined whether these expression patterns extend to other resistant and susceptible varieties.

**Fig. 3:**
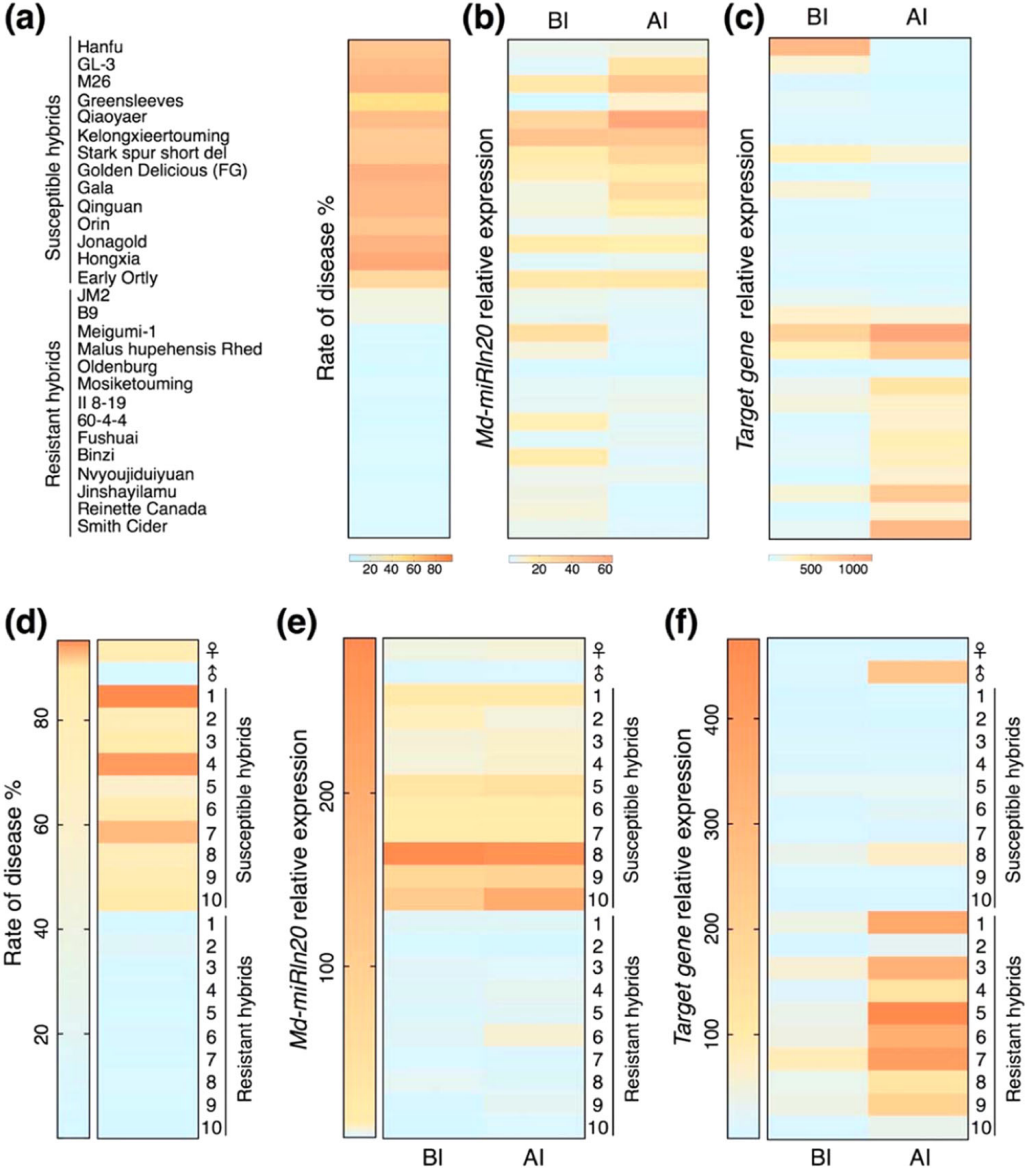
Expression pattern of *Md-miRln20* and *Target Gene* among various apple germplasms with different resistance to GLS. **a** The incidence index of apple germplasms. ‘Hanfu’, ‘GL-3’, ‘M26’, ‘Greensleeves’, ‘JM2’, ‘B9’, ‘Meigumi-1’, and *‘Malus hupehnensis* Rhed’ were tissue-cultured plantlets, and others were grown in the field. **b**, **c** Expression level of *Md-miRln20* (**b**) and *Target Gene* (**c**) in leaves before and after inoculation. **d** The incidence index of 10 susceptible and 10 resistant F_1_ hybrids of ‘Golden Delicious’ × ‘Nagafu 2’. **e**, **f** The expression level of *Md-miRln20* (**e**) and *Target Gene* (**f**) in leaves of hybrids before and after inoculation

To determine whether *Md-miRln20* expression and GLS resistance are associated in apple, expression of *miRln20* and its target was analyzed in 29 varieties and 20 F_1_ hybrids (‘Golden Delicious’ × ‘Nagafu 2’), for which disease resistance had been previously evaluated (Fig. 3a, d). *Md-miRln20* and its target gene showed different basal expression patterns in leaves of the 50 apple plants. Independent *t*-test analysis demonstrated that *Md-miRln20* expression was significantly higher (*P* < 0.01) in the leaves of 24 susceptible apple plants than in those of 25 resistant ones at 3 days after inoculation with W2 (Fig. 3b, e). The target gene was induced only in W2-infected leaves of resistant apples (Fig. 3c, f).

Thus, *Md-miRln20* expression was higher in susceptible than in resistant apple varieties, whereas the opposite was true for its target gene. Additionally, Pearson correlation coefficients between disease rates and expression levels of *Md-miRln20* and its target gene before and after inoculation were 0.28, 0.63, 0.00, and −0.63, respectively. These results suggest that the expression levels of *Md-miRln20* and its target gene after inoculation are associated with resistance to GLS in apple leaves.

### Validation of a predicted target of *Md-miRln20* by a transient GUS expression assay

**Fig. 4:**
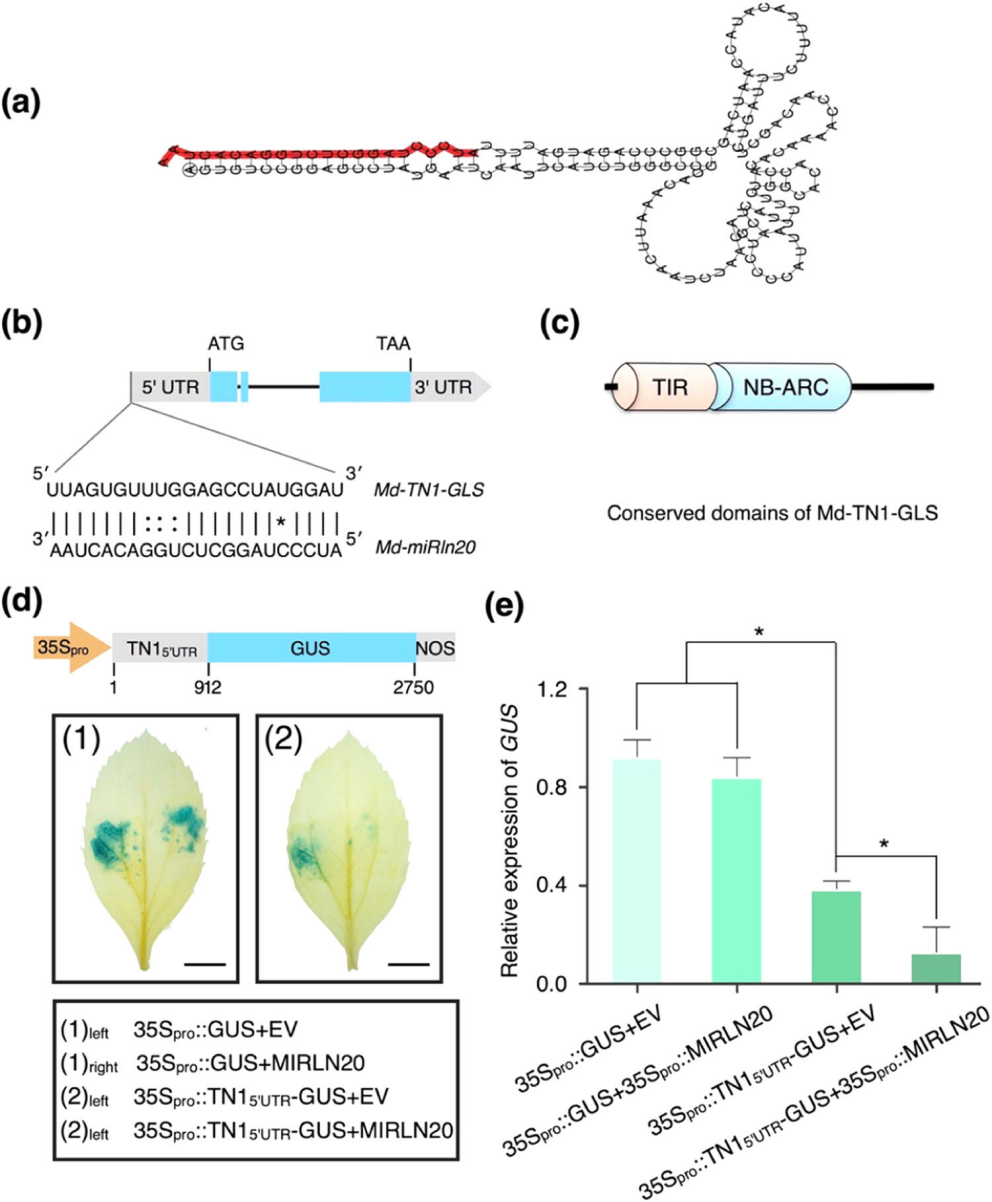
Validation of a predicted target of *Md-miRln20*. **a** Secondary structure of the miRNA precursor and the location of *Md-miRln20* (red). **b** *Md-miRln20* targets the 5′ UTR of *Md-TN1-GLS*. Blue boxes indicate exons. Lines between bases indicate Watson–Crick base pairs, dots indicate wobble base pairs, and the asterisk indicates a mismatched pair. **c** Conserved domains of Md-TN1-GLS predicted by Pfam. **d** The 5′ UTR of *Md-TN1-GLS* containing the complementarity site was fused to the *GUS* reporter gene under control of the CaMV 35S promoter (35S::TN1_5′UTR_-GUS). (1) and (2) Representative images of leaves expressing various constructs. EV, empty vector of pFGC5941; MIRLN20, overexpression of the precursor sequence of *Md-miRln20* under control of the 35S CaMV promoter. Bar = 0.4 cm. **e** Quantification of relative GUS expression levels in transformed leaves was performed in three biological replicates. Statistical significance was determined by Student’s *t*-test: *P < 0.05

We next obtained the *Md-miRln20* precursor sequence by BLAST searching the apple genome^22^ and EST libraries. Figure 4a displays the stem-loop structure of the precursor and the location of *Md-miRln20*, which is predicted to target the 5′ UTR of *MDP0000234409* (Fig. 4b). Conserved domain analysis using Pfam (http://pfam.xfam.org)^23^ showed that the protein encoded by *MDP0000234409* contains a Toll-like/interleukin-1 receptor (TIR) domain and a NBS, characteristics of the *NBS* gene family. We thus named the gene *Md-TN1-GLS* (Fig. 4c). To confirm that *Md-TN1-GLS* is regulated by *Md-miRln20*, the 5′ UTR region of *Md-TN1-GLS* containing the complementarity site was fused to a *GUS* reporter gene under control of the CaMV 35S promoter. Insertion of the 5′ UTR sequence caused downregulation of GUS in apple leaves, but when TN1_5′UTR_-GUS was transiently coexpressed with *MIRLN20*, expression of *GUS* was much lower than that in controls (Fig. 4d, e). Together with the opposite expression patterns of *Md-miRln20* and *Md-TN1-GLS* in apple leaves, these data suggest that *Md-miRln20* negatively regulates *Md-TN1-GLS* at the post-transcriptional level.

### *Md-miRln20* negatively regulates apple resistance to GLS by suppressing *Md-TN1-GLS*

**Fig. 5:**
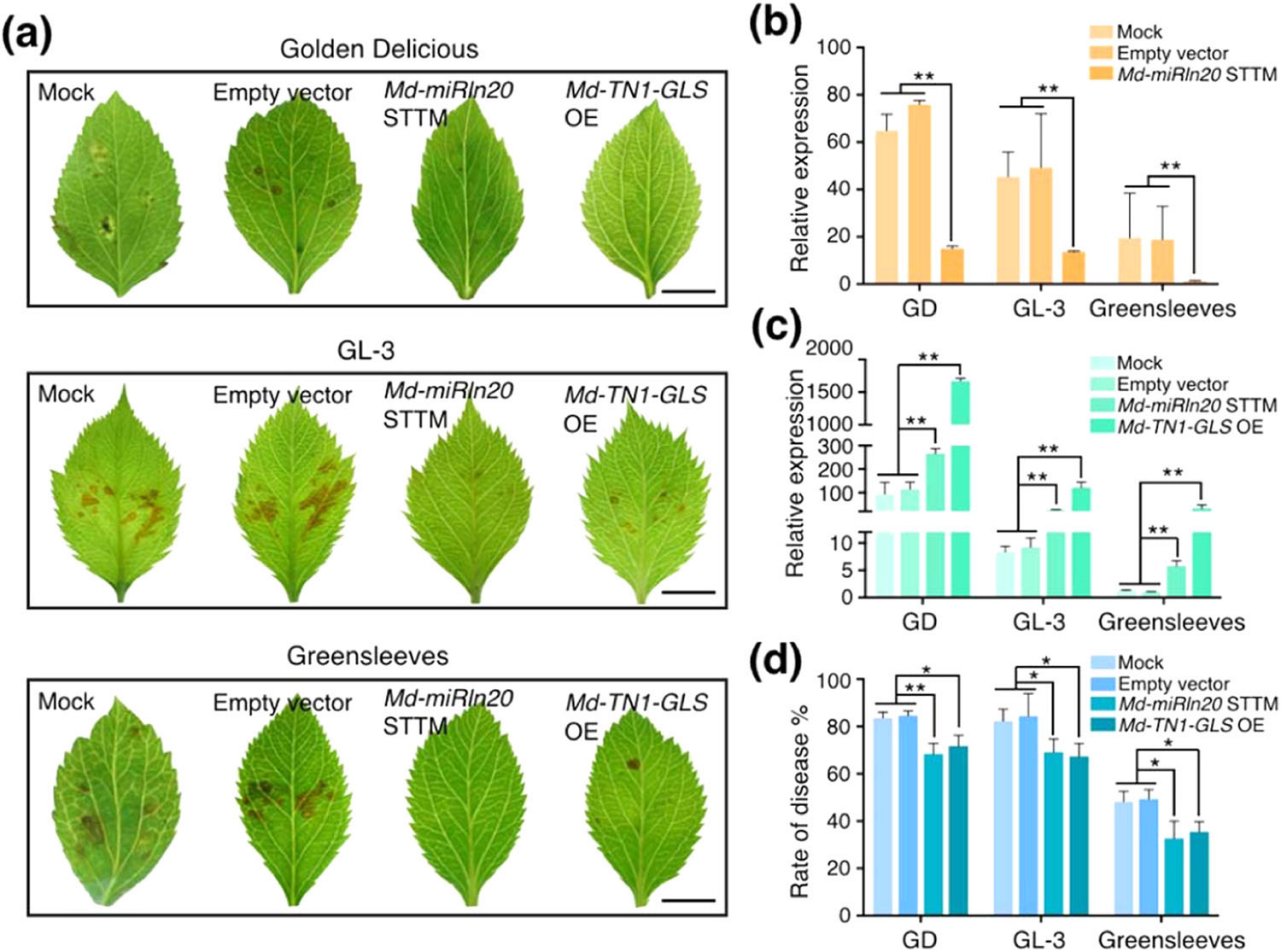
Transient expression of *Md-miRln20* and *Md-TN1-GLS* in leaves from three susceptible tissue-cultured apple varieties. **a** Symptoms of GD, ‘GL-3’, and ‘Greensleeves’ leaves at 3 DAI with W2. Bar = 0.5 cm. **b**, **c** Expression levels of *Md-miRln20* (**b**) and *Md-TN1-GLS* (**c**) in leaves expressing the indicated constructs, as analyzed by RT-qPCR. **d** Disease incidence of leaves expressing the indicated constructs. EV, empty vector of pFGC5941; Md-miRln20 STTM, overexpression of the Md-miRln20 STTM sequence under control of the 35S CaMV promoter to silence *Md-miRln20*; Md-TN1-GLS OE, overexpression of *Md-TN1-GLS* under control of the 35S CaMV promoter. Expression values are means ± SD of three biological replicates, and statistical significance was determined by Student’s *t*-test: *P < 0.05 and **P < 0.01

To investigate whether *Md-miRln20* participates in resistance to GLS, a short tandem target mimic (STTM) of *Md-miRln20*^24,25^ to silence *Md-miRln20* was cloned into the binary vector pFGC5941 and transiently expressed in the leaves of three susceptible tissue-cultured apple plantlets. RT-qPCR analysis indicated a significant decrease in the abundance of *Md-miRln20* in the leaves of GD, ‘GL-3’, and ‘Greensleeves’ at 3 days after agro-infiltration (Fig. 5b).

**Fig. 6:**
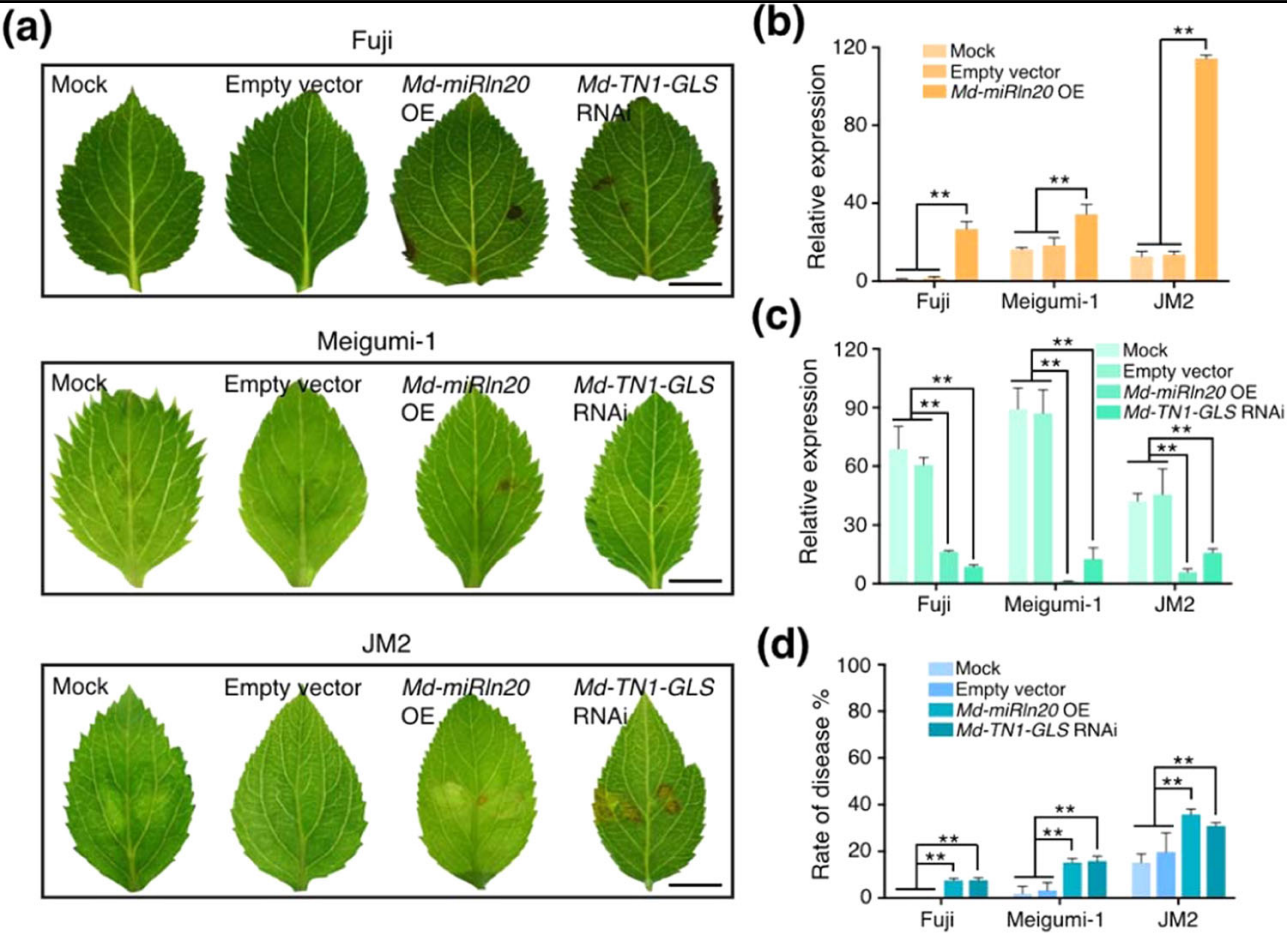
Transient expression of *Md-miRln20* and *Md-TN1-GLS* in leaves of three resistant tissue-cultured apple varieties. **a** Symptoms of ‘Fuji’, ‘Meigumi-1’, and ‘JM2’ leaves at 3 DAI with W2. Bar = 0.5 cm. **b**, **c** Expression levels of *Md-miRln20* (**b**) and *Md-TN1-GLS* (**c**) in leaves expressing the indicated constructs, as analyzed by RT-qPCR. **d** Disease incidence in leaves expressing the indicated constructs. Expression values are means ± SD of three biological replicates, and statistical significance was determined by Student’s *t*-test: *P < 0.05 and **P < 0.01

We then inoculated leaves harboring STTM-Md-miRln20 with W2 and found the mRNA level of *Md-TN1-GLS* to be increased at 3 DAI (Fig. 5c). In addition, expression of STTM-Md-miRln20 suppressed W2 infection symptoms (Fig. 5a) and significantly reduced the rate of disease (Fig. 5d), as did overexpression of *Md-TN1-GLS* (Fig. 5a, c, d). These results suggest that silencing of *Md-miRln20* increased expression of *Md-TN1-GLS*, which contributed to enhanced resistance of susceptible apples to W2. In contrast, overexpression of *MIRLN20* in ‘Fuji’, ‘Meigumi-1’, and ‘JM2’ (three resistant apple varieties) resulted in decreased expression of *Md-TN1-GLS* (Fig. 6b, c), and the leaves showed more necrotic lesions (Fig. 6a) as well as a higher disease incidence (Fig. 6d). Furthermore, silencing of *Md-TN1-GLS* rendered leaves more susceptible to W2 (Fig. 6a, c, d), as did overexpression of *Md-MIRLN20*. Therefore, *Md-miRln20* appears to regulate the resistance of apple to GLS by suppressing expression of *Md-TN1-GLS*.

## Discussion

### miRNAs participate in plant disease

An increasing number of small RNAs (sRNAs) involved in plant immunity have been identified recently^13,26,27^. In this study, we used small RNA sequencing (sRNA-seq) to identify a novel miRNA, named *Md-miRln20*, as a negative regulator of resistance to GLS in apple.

Various sRNAs play crucial roles in plant defense processes, of which small interfering RNAs (siRNAs) and miRNAs are the two major groups. sRNAs actively regulate immunity in response to different pathogens, including bacteria^28^, fungi^29^, viruses^30^, and nematodes^31^. Upon infection, up- or downregulation of sRNAs leads to changes in target gene expression. Some sRNAs facilitate plant resistance. *M. hupehensis* plants transiently overexpressing miR168 exhibited reduced sensitivity to *Botryosphaeria dothidea* infection^32^. Similarly, transgenic rice (*Oryza sativa*) plants overexpressing miR160a and miR398b displayed enhanced resistance to *Magnaporthe oryzae*, as demonstrated by decreased fungal growth, increased hydrogen peroxide accumulation at the infection site, and upregulated expression of defense-related genes^33^.

Conversely, some sRNAs negatively regulate plant pathogen resistance. In rice, miRNA528 was upregulated and cleaved *L-ASCORBATE OXIDASE* (*AO*) messenger RNA upon viral infection, thereby reducing AO-mediated accumulation of reactive oxygen species and suppressing viral resistance^34^. Three *Populous*-specific miRNAs (Ptc-miR482, Ptc-miR482-1444, and Ptc-miR482-1448) promote cleavage of polyphenol oxidase (*PPO*) transcripts as well as the transcripts of *R* genes regulating biotic and abiotic stress tolerance. Downregulation of miR482 and miR1448 in *Verticillium*-infected *Gossypium hirsutum* resulted in accumulation of both PPO and disease resistance proteins^35^. In the present study, overexpression of a novel miRNA, *Md-miRln20*, resulted in susceptibility to GLS in apple leaves through suppression of the corresponding target gene *Md-TN1-GLS* (Fig. 6).

In addition, some sRNAs act as mobile signals that mediate cross-kingdom RNA interference (RNAi) during host–pathogen interactions^36^. In response to infection with *V. dahliae*, cotton plants showed increased production of miR166 and miR159 and exported both to the hyphae of this fungus to silence two essential fungal virulence genes^37^. Pst-milR1 is a novel miRNA-like RNA in *Puccinia striiformis f. sp. tritici* that acts as an effector that suppresses host immunity by binding to the transcripts of *Triticum aestivum pathogenesis-related gene 2* (*PR2*)^38^. These findings suggest that cross-kingdom RNAi is likely to exist and that such a situation is worthy of exploration in the apple–*G*. *cingulata* interaction, the resistance mechanism of which remains unclear.

### The contribution of *Md-TN1-GLS* to GLS resistance in apples is likely regulated by SA

*Md-TN1-GLS* is the target gene of *Md-miRln20*, and its expression promotes resistance to GLS in apple. Md-TN1-GLS contains a TIR domain and an NBS domain and belongs to the *NBS* gene family (Fig. 4c). NBS receptors are the most commonly known plant R proteins that detect effectors from pathogens. They are classified into two groups based on their N-terminal domains: Toll-like/interleukin-1 receptor (TIR)-type NBS proteins and coiled-coil (CC)-type NBS proteins^39^. TIR-NB (TN) proteins have also been identified in plants, containing TIR and NBS domains but lacking LRRs^40^. Md-TN1-GLS is a newly identified TN protein in apple that was upregulated in resistant apple leaves upon W2 infection. Overexpression of *Md-TN1-GLS* in susceptible apple varieties increased their resistance to GLS, whereas downregulation rendered apples more susceptible (Figs. 5 and 6). These results indicate that *Md-TN1-GLS* contributes to apple resistance to GLS.

NBS receptors trigger local resistance associated with programmed cell death as part of the hypersensitive response and also amplify basal defenses involving the signaling hormone salicylic acid (SA), leading to systemic resistance^41^. *Arabidopsis* lines stably overexpressing *TX* and *TN* genes display a variety of phenotypes associated with basal innate immune responses, which are associated with elevated levels of SA^42^. Pretreatment with SA strongly induces resistance to GLS in ‘Gala’ apple leaves, with SA-treated leaves showing a significant reduction in lesion number and disease index^3^. We analyzed the promoter sequences of *Md-TN1-GLS* in PlantCARE (http://bioinformatics.psb.ugent.be/webtools/plantcare/html/) and identified several *cis*-acting elements involved in SA responsiveness (data not shown). Overall, upregulation of *Md-TN1-GLS* in resistant apple leaves is likely regulated by SA, though this remains to be confirmed.

### Candidate genes influencing GLS resistance in apple

It has been reported that GLS resistance is controlled by a single recessive gene (*R*_*gls*_) in apple^6^. The locus is on chromosome 15, sandwiched between the markers SNP4208 and SNP4257, with a recombination frequency of 0.97%^6,7^. Nine candidate genes are predicted in the 49-kb genetic interval between SNP4208 and SNP4257^7^; however, the recessive major gene remains to be identified.

In this work, *Md-miRln20* was found to negatively regulate resistance to GLS by suppressing expression of *Md-TN1-GLS* in apple. BLAST results showed that *Md-TN1-GLS* is located on chromosome 2 in a region that is homeologous to the central region of Chromosome 15 ^43^. However, we found no *Md-TN1-GLS-like* gene in the region containing *R*_*gls*_ and tightly homeologous to Chromosome 2, where *Md-TN1-GLS* is located. These results indicate that resistance to GLS might be modified by several other genes, such as *Md-TN1-GLS*, in addition to the recessive major gene. Further molecular studies are underway to investigate this possibility.

In conclusion, we found that the expression levels of *Md-miRln20* and its target gene correlate with resistance to GLS in apples. Furthermore, downregulating *Md-miRln20* or overexpressing *Md-TN1-GLS* in susceptible apple leaves reduced the rate of disease, whereas overexpressing *Md-miRln20* or silencing *Md-TN1-GLS* in resistant apple leaves increased the disease incidence. Our study demonstrates that *Md-miRln20* negatively regulates resistance to GLS by suppressing expression of *Md-TN1-GLS* in apple.

## Materials and methods

### Plant materials and growth conditions

Tissue-cultured *Malus*×*domestica* Borkh. cv. ‘Golden Delicious’ and ‘Fuji’ plantlets were grown on Murashige and Skoog (MS) medium^44^ containing 0.6 mg L^−1^ 6-benzylaminopurine (6-BA) and 0.15 mg L^−1^ 1-naphthylacetic acid (NAA) in a climate-controlled culture room at 25 ± 1 °C with a 16/8-h light/dark photoperiod. The plants were transferred to fresh medium every 4 weeks.

Twenty apple varieties, 7–8 years old, were grown at the National Germplasm Repository of Apple (Institute of Pomology of Chinese Academy of Agricultural Sciences, CAAS, Xingcheng, Liaoning Province, China).

The crossing parents ‘Golden Delicious’, ‘Nagafu 2’, ‘Hanfu’, ‘Yueshuai’, ‘62-45’, and ‘Ryoka no kisetsu’ were 15-year-old trees, and the three hybrid groups were 6 years old and grown at the Liaoning Institute of Pomology.

### Fungal growth, infection assay, and statistical analysis of disease incidence

The highly pathogenic fungal strain W2 (*G. cingulata*) was isolated by Professor Zongshan Zhou from the Institute of Pomology of CAAS. The W2 monospore culture was maintained on potato dextrose agar (PDA). Conidia were produced on PDA according to a method described previously^3^. A concentration of 1 × 10^5^ conidia mL^−1^, which was assessed by microscopy (Olympus, CX31RTSF, Japan), was used to inoculate detached apple leaves by dropping 10 μL suspensions onto a unwounded abaxial surface.

For tissue-cultured plantlets, after 4 weeks of culture, expanded leaves of similar size were inoculated on the unwounded abaxial surface using 10 μL of conidia suspensions (1 × 10^5^ conidia mL^−1^). For field-grown trees, 5-cm-long leaves were collected, disinfected with 75% (v/v) ethanol, placed on moist filter paper in a culture dish with the base of the petiole wrapped in a moist cotton ball, and incubated under the same conditions as the tissue culture plantlets (humidity, 75%; temperature, 25 °C). Necrotic lesions on each leaf were visually examined 3 days after inoculation (DAI), and disease incidence was recorded. The experiments were repeated three times, and *t*-test analysis was performed. Three biological replicates of approximately 50 leaves from different tissue-cultured plantlets were conducted to reduce experimental error.

### RNA extraction, library construction, and sequencing

Mixed leaves of ‘Golden Delicious’ inoculated with W2 (*G. cingulata* (Stoneman) Spauld. & H. Schrenk) were collected at 3 DAI, and total RNA was isolated using the modified cetyltrimethyl ammonium bromide (CTAB) method^45^. RNA samples with high purity (OD 260/280 between 1.8 and 2.2) and integrity (RNA integrity number of 6.5 or higher) were used for small RNA library preparation and sequencing by Tianjin Biochip Corporation (Tianjin, China). Small RNA fragments (18–30 nucleotides) were purified on a 15% denaturing polyacrylamide gel and ligated with 3′ and 5′ adaptors. The ligated products were used for cDNA synthesis, followed by acrylamide gel purification and PCR amplification to generate small RNA libraries. An Agilent 2100 Bioanalyzer (Agilent, USA) was used for quantification and qualification of the sample libraries. The libraries were sequenced using the Illumina HiSeq 2500 sequencing platform (Illumina Inc., San Diego, CA, USA).

### Sequence data analysis

The raw reads obtained by RNA sequencing (RNA-seq) were filtered by removing duplication sequences, low-quality reads, reads smaller than 18 nt, adaptor sequences, and contamination formed via adaptor–adaptor ligation. Other RNAs (rRNA, tRNA, snRNA, and snoRNA) were removed by BLAST searching against the GenBank database (http://blast.ncbi.nlm.nih.gov) and Rfam database (http://rfam.xfam.org/)^21,46^. The remaining clean reads were used for detecting conserved and novel miRNAs.

### Identification of conserved and putative novel miRNAs and target prediction

To detect conserved and novel miRNAs, clean reads from the W2-inoculated library were searched against known plant miRNAs in miRBase 21.0 (www.mirbase.org/) with a maximum of three mismatches allowed to identify conserved miRNAs^20^. Sequences that did not align to any database entries were considered novel putative miRNAs. miREvo^47^ and mirdeep2^48^ software programs were used to predict novel miRNAs that mapped to the apple genome v3.0.a1^22^ (https://www.rosaceae.org/species/malus/all). The resulting sequences were screened for the presence of stem-loop secondary structures using mfold software^49^.

To predict miRNA target genes, miRNA sequences were aligned to the apple genome using Plant Small RNA Target Analysis Server (http://plantgrn.noble.org/psRNATarget/). The predicted target genes were evaluated based on complementarity and maximum expectation, as previously described^50^. The functions of the putative target genes were analyzed using Genome Database for Rosaceae^22,43^ (https://www.rosaceae.org) and the National Center for Biotechnology Information genomic database (http://www.ncbi.nlm.nih.gov).

### Expression analysis of miRNAs and target genes in response to GLS by RT-qPCR

Leaves were obtained from plants at 3 DAI with W2 or water, frozen in liquid nitrogen, and stored at −80 °C until use. Total RNA was extracted using a modified CTAB method^45^. An ND-1000 NanoDrop spectrophotometer (Thermo Fisher Scientific, USA) was utilized to measure RNA concentrations. DNase-treated RNA (1 μg) was used for the reverse transcription reactions with miRcute Plus miRNA First-Strand cDNA Synthesis Kit (Tiangen Biotech Co., Ltd., China) or M-MLV Reverse Transcriptase (Promega, America). Reverse transcription-quantitative PCR (RT-qPCR) was performed using miRcute plus miRNA Premix and SuperReal PreMix Plus (Tiangen Biotech Co., Ltd., China). Thermocycling involved a program of 40 cycles of 95 °C for 10 s and 60 °C for 30 s with a StepOnePlus Real-Time PCR System (Thermo Fisher Scientific, USA). Relative expression of the miRNAs and target genes was determined using the 2^−ΔΔCt^ method^46^. *U6* was used as the internal reference for miRNAs^13^, and *MdActin* was used as the reference for target genes^46^. All RT-qPCR analyses were performed in three biological replicates, each of which consisted of three technical replicates. Gene-specific primers are provided in Table S6.

### Target validation by transient GUS expression assay

To generate the TN1s_5′UTR_-GUS construct, the 912-kb genomic sequence upstream of *Md-TN1-GLS* was cloned and ligated into pCAMBIA1305.1 (TransGen Biotech, China) between the CaMV 35S promoter and *GUS* gene. For expression of *MdmiRln20*, the precursor sequences obtained by PCR were ligated into the pFGC5941 vector. All of the constructs were transformed into *Agrobacterium tumefaciens* strain GV3101 by heat-shock transformation, and transient GUS expression assays were carried out by injecting *Agrobacterium* cells into the leaves of 4-week-old tissue-cultured ‘Golden Delicious’, as previously described^13^. The infiltrated plantlets were cultured on MS culture medium in a climate-controlled room at 25 ± 1 °C with a 16/8-h light/dark photoperiod. Leaves were collected for GUS staining and gene expression analysis at 3 days after *Agrobacterium* infiltration. GUS staining was performed as previously described^13^.

### Functional analysis of *MdmiRln20* and its target gene by transient expression in apple

STTMs technology-based vectors were used to block endogenous *MdmiRln20* activity^24^. Briefly, the consensus trinucleotide bulge sequence CTA was inserted between the 11th and 12th position from the 3′ end of the reverse complementary sequences of mature miRNAs to generate small RNA-binding (SRB) sequences. Tandem repeats of these SRB sequences were interspersed by a 48-nt imperfect stem-loop linker (ISLL; 5′-GTTGTTGTTGTTATGGTCTAATTTAAATATGGTCTAAAGAAGAAGAAT-3′)^46^. The STTM sequences were synthesized and cloned into the pFGC5941 vector. To generate the *Md-TN1-GLS* overexpression vector, the full-length coding sequences were cloned into pFGC5941. For RNA interference of *Md-TN1-GLS*, a partial unique sequence (5′-AAAAGTTCAAAATTTGAAGACAACAAGGAGAAGATCCTCACATGGAGGAGTGCTCTTACAGATGCAGCAAGTTTGTCAGGATATACTTTCAAAGAGGGAGAGTATGAAGCTACATTTATCAGTAAGATCGTGGAGGAGATCTTCGT-3′) was ligated to the 5′ end of the intron in pFGC5941, and the reverse-complement sequence was ligated to the 3′ end of the intron to produce siRNAs. All of the constructs were transformed into *Agrobacterium* strain GV3101 by heat-shock transformation^46^. Transformed *Agrobacterium* cells were injected into the leaves of 4-week-old tissue-cultured ‘Golden Delicious’, ‘GL-3’, ‘Greensleeves’, ‘Fuji’, ‘Meigumi-1’, or ‘JM2’, as previously described^46^. The infiltrated plantlets were cultured on MS culture medium in a climate-controlled room at 25 ± 1 °C with a 16/8-h light/dark photoperiod. Leaves were inoculated with W2 at 2 days after *Agrobacterium* infiltration, and the disease incidence was recorded at 3 DAI. Three leaves from three different plantlets were collected as a sample for gene expression analysis.

### DNA extraction and SSR analysis

Leaves from hybrids of ‘Golden Delicious’ × ‘Nagafu 2’, ‘Hanfu’ × ‘Yueshuai’, and ‘62-45’ × ‘Ryoka no kisetsu’ were ground for DNA extraction using a modified CTAB method^13^. Simple sequence repeat (SSR) analysis was carried out by PCR using a previously screened pair of primers (S0405127)^8^. The SSR PCR reaction was performed in a 15-μL volume, which consisted of 2 μL of genomic DNA (10 ng μL^−1^), 7.5 μL of 2× Master Mix (Cwbiotech, Beijing, China), and 0.8 μL of each primer (10 μM). The conditions for PCR amplification were as follows: 94 °C for 5 min, 34 cycles of 95 °C for 30 s, 54 °C for 30 s, and 72 °C for 20 s, followed by 72 °C for 10 min. The amplified products were separated on 2.5% agarose gels.

## Supplementary information

Supplementary Fig. S1-S3 and supplementary Tables S1-S6.
