# Supplementary material for: A novel miRNA negatively regulates resistance to Glomerella leaf spot by suppressing expression of an NBS gene in apple
Source: Hortic Res. 2019 Aug 1;6:93. doi: 10.1038/s41438-019-0175-x (PMC6804642; doi:10.1038/s41438-019-0175-x)
Supplement: Supplementary file 1 — Supplementary Fig. S1-S3 and supplementary Tables S1-S6. [file 41438_2019_175_MOESM1_ESM.docx]

**A novel miRNA negatively regulates resistance to Glomerella leaf spot by suppressing expression of an *NBS* gene in apple**

Yi Zhang, Qiulei Zhang, Li Hao, Shengnan Wang, Shengyuan Wang, Wenna Zhang, Chaoran Xu, Yunfei Yu, Tianzhong Li*

**SUPPLEMENTARY DATA**


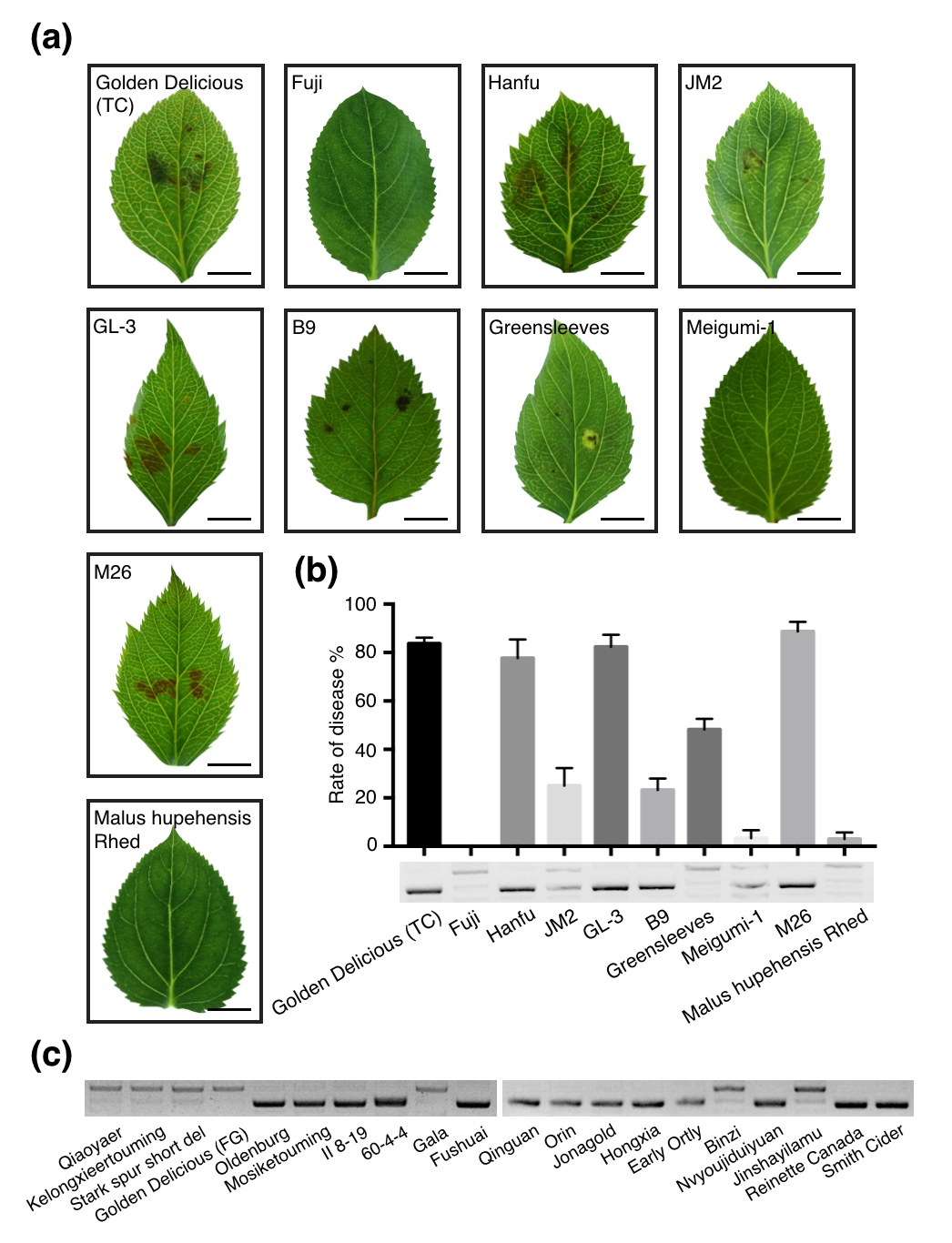


Figure S1. Resistance evaluation of apple germplasms to Glomerella Leaf Spot.

(a) Symptoms of leaves from 10 tissue-cultured (TC) apples 3 DAI with *G. cingulata*. Bar = 0.3 cm. (b) Disease rates and SSR (S0405127) analysis of the 10 apple varieties. The smaller band was 330 bp and represented the susceptible varieties. (c) SSR (S0405127) analysis of 20 field-grown (FG) apple varieties.


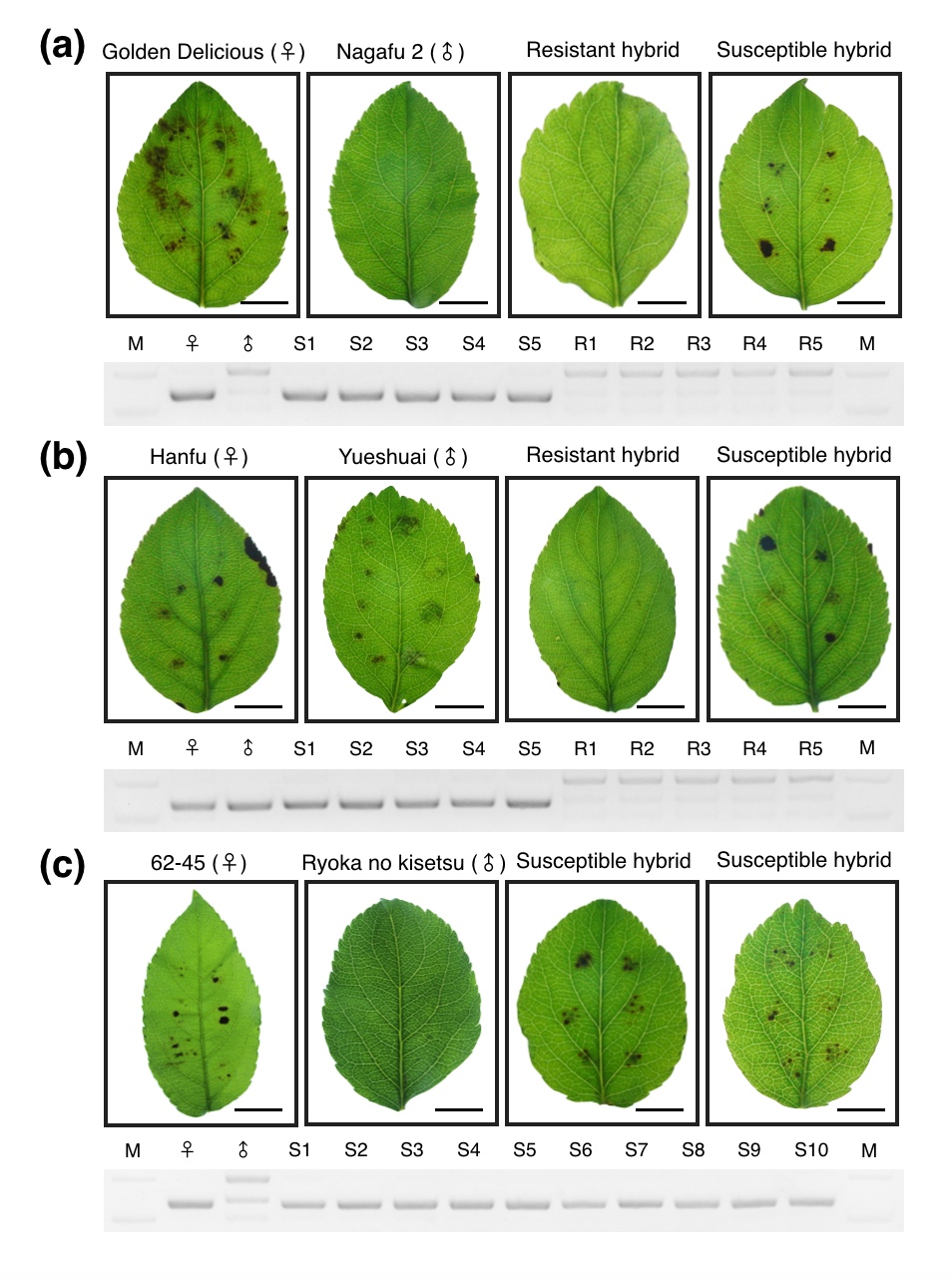


Figure S2. Resistance evaluation and SSR (S0405127) analysis of a portion of the three F1 hybrid groups.

(a) Ten F1 hybrids of 'Golden Delicious' × 'Nagafu 2'. (b) Ten F1 hybrids of 'Hanfu' × 'Yueshuai'. (c) Ten F1 hybrids of '62-45' × 'Ryoka no kisetsu'. Bar = 1.5 cm.


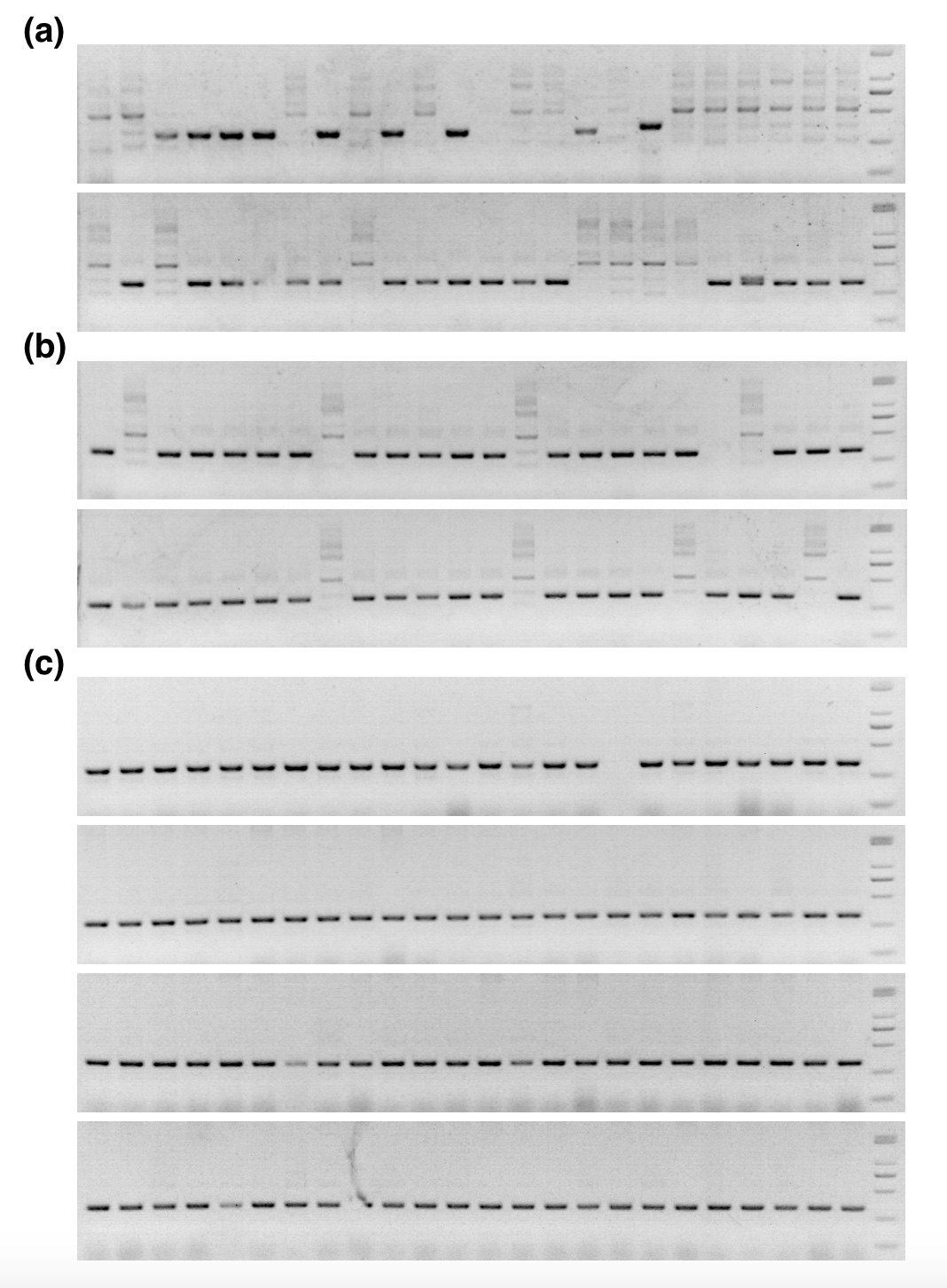


Figure S3. SSR (S0405127) analysis of the three F1 hybrid groups.

(a) Forty-eight F1 hybrids of 'Golden Delicious' × 'Nagafu 2'. (b) Forty-eight F1 hybrids of 'Hanfu' × 'Yueshuai'. (c) Ninety-six F1 hybrids of '62-45' × 'Ryoka no kisetsu'.

Table S1. Apple germplasms used in this work.

| **Variety** | **Source** | **Plant type** |
| --- | --- | --- |
| Golden Delicious (TC) | Wanmei Jin Lab from Beijing Academy of Agriculture and Forestry Sciences | Tissue-cultured |
| Fuji | Established in our lab | Tissue-cultured |
| Hanfu | Established in our lab | Tissue-cultured |
| JM2 | Established in our lab | Tissue-cultured |
| GL-3 | Zhihong Zhang Lab from Shenyang Agricultural University | Tissue-cultured |
| B9 | Established in our lab | Tissue-cultured |
| Greensleeves | Established in our lab | Tissue-cultured |
| Meigumi-1 | Established in our lab | Tissue-cultured |
| M26 | Established in our lab | Tissue-cultured |
| Malus hupehensis Rhed | Wanmei Jin Lab from Beijing Academy of Agriculture and Forestry Sciences | Tissue-cultured |
| Qiaoyaer | Institute of Pomology of Chinese Academy of Agricultural Sciences | Field-grown |
| Kelongxieertouming | Institute of Pomology of Chinese Academy of Agricultural Sciences | Field-grown |
| Stark spur short del | Institute of Pomology of Chinese Academy of Agricultural Sciences | Field-grown |
| Oldenburg | Institute of Pomology of Chinese Academy of Agricultural Sciences | Field-grown |
| Mosiketouming | Institute of Pomology of Chinese Academy of Agricultural Sciences | Field-grown |
| II 8-19 | Institute of Pomology of Chinese Academy of Agricultural Sciences | Field-grown |
| 60-4-4 | Institute of Pomology of Chinese Academy of Agricultural Sciences | Field-grown |
| Gala | Institute of Pomology of Chinese Academy of Agricultural Sciences | Field-grown |
| Fushuai | Institute of Pomology of Chinese Academy of Agricultural Sciences | Field-grown |
| Qingguan | Institute of Pomology of Chinese Academy of Agricultural Sciences | Field-grown |
| Orin | Institute of Pomology of Chinese Academy of Agricultural Sciences | Field-grown |
| Jonagold | Institute of Pomology of Chinese Academy of Agricultural Sciences | Field-grown |
| Hongxia | Institute of Pomology of Chinese Academy of Agricultural Sciences | Field-grown |
| Early Ortly | Institute of Pomology of Chinese Academy of Agricultural Sciences | Field-grown |
| Binzi | Institute of Pomology of Chinese Academy of Agricultural Sciences | Field-grown |
| Nvyoujiduiyuan | Institute of Pomology of Chinese Academy of Agricultural Sciences | Field-grown |
| Jinshayilamu | Institute of Pomology of Chinese Academy of Agricultural Sciences | Field-grown |
| Reinette Canada | Institute of Pomology of Chinese Academy of Agricultural Sciences | Field-grown |
| Smith Cider | Institute of Pomology of Chinese Academy of Agricultural Sciences | Field-grown |
| Golden Delicious (FG) | Liaoning Institute of Pomology | Field-grown |
| Nagafu 2 | Liaoning Institute of Pomology | Field-grown |
| Hanfu | Liaoning Institute of Pomology | Field-grown |
| Yueshai | Liaoning Institute of Pomology | Field-grown |
| 62-45 | Liaoning Institute of Pomology | Field-grown |
| Ryoka no kisetsu | Liaoning Institute of Pomology | Field-grown |
| Hybrids | Liaoning Institute of Pomology | Field-grown |

Table S2. Statistical analysis of the separation ratio in three F1 hybrid groups.

| **Cross combination** | **Total hybrids** | **Susceptible hybrids** | **Resistant hybrids** | **Ratio of S to R** | **Expected ratio** | ***χ2*** |
| --- | --- | --- | --- | --- | --- | --- |
| Golden Delicious × Nagafu 2 | 47 | 26 | 21 | 1.18 : 1 | 1 : 1 | 0.34 |
| Hanfu × Yueshuai | 47 | 39 | 8 | 4.75 : 1 | 3 : 1 | 1.20 |
| 62-45 × Ryoka no kisetsu | 95 | 95 | 0 | 95 : 0 | 1 : 0 | 0.00 |

Table S3. Sequencing read statistics of the small RNA libraries constructed from inoculated 'Golden Delicious' leaves.

| **Sample** | **total reads** | **N% > 10%** | **low**  **quality** | **5' adapter contamine** | **3' adapter null or insert null** | **with ploy**  **A／T／G／C** | **clean reads** |
| --- | --- | --- | --- | --- | --- | --- | --- |
| GD-W2 | 10096436  （100%） | 6  （0.00%） | 42793  （0.42%） | 7485  （0.07%） | 272597  （2.70%） | 20667  （0.20%） | 9752888  （96.60%） |

Table S4. A portion of the predicted target genes of known miRNAs found in inoculated 'Golden Delicious' leaves.

| **miRNA** | **Gene ID** | **Target Description** |
| --- | --- | --- |
| Md-miR1511 | MDP0000138140 | Probable magnesium transporter NIPA3 |
| Md-miR156p | MDP0000274727 | putative two-component response regulator ARR13 |
| Md-miR156x | MDP0000778465 | Squamosa promoter-binding-like protein 6 |
| Md-miR159a | MDP0000123466 | Auxin response factor 23-like |
| Md-miR159c | MDP0000124536 | Transcription factor bHLH91-like |
| Md-miR160a | MDP0000750392 | Auxin response factor 18-like |
| Md-miR162a | MDP0000229637 | Uncharacterized protein |
| Md-miR164a | MDP0000911724 | ARATH NAC domain-containing protein 100-like |
| Md-miR166a | MDP0000126553 | Homeobox-leucine zipper protein ATHB-8-like |
| Md-miR167b | MDP0000550049 | Auxin response factor 8 |
| Md-miR171c | MDP0000151144 | Scarecrow-like protein 6 |
| Md-miR171f | MDP0000274120 | Scarecrow-like protein 27 |
| Md-miR171g | MDP0000209022 | Synaptotagmin-5-like |
| Md-miR171i | MDP0000429896 | Gibberellin-regulated protein 12-like |
| Md-miR171m | MDP0000151144 | Scarecrow-like protein 6 |
| Md-miR172i | MDP0000935652 | Probable WRKY transcription factor 31 |
| Md-miR2111a | MDP0000892979 | F-box/kelch-repeat protein At3g27150-like |
| Md-miR2118a | MDP0000184039 | Serine/threonine-protein kinase D6PK-like |
| Md-miR319a | MDP0000916623 | Transcription factor TCP4-like |
| Md-miR3627a | MDP0000237183 | Protein FIZZY-RELATED 1-like |
| Md-miR393a | MDP0000469943 | Protein AUXIN SIGNALING F-BOX 2-like |
| Md-miR394a | MDP0000853426 | Transcription factor WER-like |
| Md-miR396a | MDP0000619907 | Dicer-like protein 4 |
| Md-miR396b | MDP0000237091 | Agamous-like MADS-box protein AGL62 |
| Md-miR396f | MDP0000183534 | Dof zinc finger protein DOF4.6-like |
| Md-miR398b | MDP0000375032 | Blue copper protein-like |
| Md-miR399a | MDP0000206582 | Disease resistance protein RPP5-like |
| Md-miR399e | MDP0000206300 | Cell wall / vacuolar inhibitor of fructosidase 1-like |
| Md-miR403a | MDP0000260407 | Protein argonaute 2-like |
| Md-miR408a | MDP0000300452 | Lysine histidine transporter-like 8 |
| Md-miR408b | MDP0000307124 | Short-chain type dehydrogenase/reductase-like |
| Md-miR477a | MDP0000204905 | Uncharacterized protein |
| Md-miR535a | MDP0000166392 | Peroxisomal membrane protein PEX14-like |
| Md-miR535b | MDP0000286458 | Two-component response regulator-like APRR1 |
| Md-miR535d | MDP0000185769 | Cysteine proteinase RD19A |
| Md-miR7120a | MDP0000288808 | B3 domain-containing transcription factor NGA1 |
| Md-miR7121a | MDP0000517257 | Ethylene-responsive transcription factor 2-like |
| Md-miR7121d | MDP0000483490 | TMV resistance protein N-like |
| Md-miR7122a | MDP0000297646 | Ethylene-responsive transcription factor ERF003-like |
| Md-miR7124a | MDP0000309729 | WD repeat-containing protein 76-like |
| Md-miR7125 | MDP0000729984 | Cinnamoyl-CoA reductase 1 |
| Md-miR7126 | MDP0000932888 | Uncharacterized protein |
| Md-miR7127a | MDP0000203957 | Uncharacterized protein |
| Md-miR827 | MDP0000418846 | Protein ABC transporter 1, mitochondrial |
| Md-miR828a | MDP0000931057 | Transcription factor WER-like |

Table S5. A portion of the predicted target genes of novel miRNAs found in inoculated 'Golden Delicious' leaves.

| **miRNA** | **Gene ID** | **Target Description** |
| --- | --- | --- |
| Md-miRln1 | MDP0000230224 | Ribonucleoside-diphosphate reductase small chain A-like |
| Md-miRln2 | MDP0000277868 | TMV resistance protein N-like |
| Md-miRln3 | MDP0000309351 | NAC domain-containing protein 73-like |
| Md-miRln4 | MDP0000149979 | Malate dehydrogenase, chloroplastic-like |
| Md-miRln5 | MDP0000729050 | Zinc finger MYM-type protein 1-like |
| Md-miRln6 | MDP0000160640 | DeSI-like protein At4g17486 |
| Md-miRln7 | MDP0000193401 | 7-deoxyloganetin glucosyltransferase-like |
| Md-miRln8 | MDP0000935996 | Probable WRKY transcription factor 26 |
| Md-miRln9 | MDP0000222500 | Elongator complex protein 4 |
| Md-miRln10 | MDP0000272708 | Protein argonaute 4 |
| Md-miRln11 | MDP0000372290 | L-type lectin-domain containing receptor kinase IV.1-like |
| Md-miRln12 | MDP0000231493 | BTB/POZ domain-containing protein At3g50780-like |
| Md-miRln13 | MDP0000319618 | Isocitrate dehydrogenase [NAD] regulatory subunit 1, mitochondrial |
| Md-miRln14 | MDP0000610304 | Probable WRKY transcription factor 16 |
| Md-miRln15 | MDP0000147309 | Transcription factor GAMYB-like |
| Md-miRln16 | MDP0000058031 | Phosphatidylinositol-3-phosphatase myotubularin-1-like |
| Md-miRln17 | MDP0000320656 | Calmodulin-binding transcription activator 3-like |
| Md-miRln18 | MDP0000204347 | MAG2-interacting protein 2-like |
| Md-miRln19 | MDP0000285780 | Transcription factor DYT1-like |
| Md-miRln20 | MDP0000234409 | TMV resistance protein N-like |
| Md-miRln21 | MDP0000183558 | NAC domain-containing protein 14-like |
|  | MDP0000298793 | Subtilisin-like protease SBT1.5 |
| Md-miRln22 | MDP0000234893 | Probable receptor-like protein kinase At1g80640-like |
| Md-miRln23 | MDP0000165902 | GPI mannosyltransferase 2-like |
| Md-miRln24 | MDP0000203957 | Uncharacterized protein |
| Md-miRln25 | MDP0000168871 | Probable WRKY transcription factor 14 |
| Md-miRln26 | MDP0000515395 | Probable leucine-rich repeat receptor-like protein kinase At1g35710 |
| Md-miRln27 | MDP0000138851 | Probable indole-3-pyruvate monooxygenase YUCCA10 |
| Md-miRln28 | MDP0000178669 | Histone deacetylase 5-like |
| Md-miRln29 | MDP0000310092 | Putative disease resistance protein At1g52660 |
| Md-miRln30 | MDP0000184270 | Disease resistance protein RPM1-like |
| Md-miRln31 | MDP0000267131 | Conserved oligomeric Golgi complex subunit 3-like |
| Md-miRln32 | MDP0000681336 | NEDD8-activating enzyme E1 regulatory subunit-like |
| Md-miRln33 | MDP0000382588 | Probable disease resistance protein At5g66910 |
| Md-miRln34 | MDP0000214891 | TMV resistance protein N-like |
| Md-miRln35 | MDP0000189652 | Signal recognition particle receptor subunit beta-like |
| Md-miRln36 | MDP0000267131 | Conserved oligomeric Golgi complex subunit 3-like |
| Md-miRln37 | MDP0000222221 | Pentatricopeptide repeat-containing protein At2g03880, mitochondrial-like |
| Md-miRln38 | MDP0000138851 | Probable indole-3-pyruvate monooxygenase YUCCA10 |
| Md-miRln39 | MDP0000164694 | Uncharacterized membrane protein At1g16860-like |
| Md-miRln40 | MDP0000320656 | Calmodulin-binding transcription activator 3-like |

Table S6. Gene-specific primers used for RT-qPCR.

| **Gene name** | **Primer** | **Sequence 5'-3'** |
| --- | --- | --- |
| Md-miRln2 | Forward | TCCTTTGATGGGAGAGATCTGG |
| Md-miRln8 | Forward | CTGAAGTGTTTGGGGGAACCC |
| Md-miRln14 | Forward | GTTTGGGAAACTGCAAAAGGAC |
| Md-miRln20 | Forward | CATCCCTAGGCTCTGGACACTAA |
| Md-miRln29 | Forward | CGTCTTTCCTAACCCTCCCATTCC |
| Md-miRln30 | Forward | AGGTGCAGGTGCCAGTGCA |
| Md-miRln33 | Forward | CGGTATGAGTGGTTGTAGAGAGTC |
| Md-miRln34 | Forward | CGGTTCCACAGCTTTCTTGAACTT |
| U6 | Forward | GGGGACATCCGATAAAATT |
| MDP0000277868 | Forward | GCATGGTGTTTGGATGGGCTTAG |
|  | Reserve | CCCTCCATCTCTGCACCTTCTC |
| MDP0000935996 | Forward | CCAGCAGCCTTCACTTTCTCAAC |
|  | Reserve | AGGCGGCGGTAGTGACTTG |
| MDP0000610304 | Forward | TATGGTTGCGTTGGTTTGGATGC |
|  | Reserve | AGATTCTGGCAGCCTTGGAGC |
| MDP0000234409 | Forward | GATGGTGCTTGGATGAGCTTGTAC |
|  | Reserve | CATACTCTCCCTCTTTGAAAGTATATCCTG |
| MDP0000310092 | Forward | ATTGTTGGTGGAAGTGGATGCCTTG |
|  | Reserve | ACCTCATCAAACATCGACTCCATCC |
| MDP0000184270 | Forward | GCGAGAGAGGCAGATGAGGAG |
|  | Reserve | CAAAGGAGGTGCAGATGGCAATG |
| MDP0000382588 | Forward | AGGGCGTAAGGGATGCGAG |
|  | Reserve | AACGGCAAATCCAACCCGAC |
| MDP0000214891 | Forward | CACAAGTGGAGGGCTGCTTTGAC |
|  | Reserve | TTTCTTGACTAAATCAGCCTCCCTCC |
| S0405127 | Forward | GGCACAATGTAGGAGGGATA |
|  | Reserve | GCTATGAGGAAATTGGCTCT |
